# Synthesis, analysis of mol­ecular and crystal structures, estimation of inter­molecular inter­actions and biological properties of 1-benzyl-6-fluoro-3-[5-(4-methylcyclohexyl)-1,2,4-oxadiazol-3-yl]-7-(piperidin-1-yl)quinolin-4-one

**DOI:** 10.1107/S2056989023001305

**Published:** 2023-02-21

**Authors:** Yevhenii Vaksler, Halyna V. Hryhoriv, Vladimir V. Ivanov, Sergiy M. Kovalenko, Victoriya A. Georgiyants, Thierry Langer

**Affiliations:** a SSI "Institute for Single Crystals", National Academy of Sciences of Ukraine, 60 Nauky Ave, Kharkiv 61001, Ukraine; b The National University of Pharmacy, 53 Pushkinska St, Kharkiv 61002, Ukraine; c V. N. Karazin Kharkiv National University, 4 Svobody Sq., Kharkiv 61077, Ukraine; d Department of Pharmaceutical Chemistry, University of Vienna, Althanstrabe 14, A-1090, Vienna, Austria; Indian Institute of Science Education and Research Bhopal, India

**Keywords:** mol­ecular structure, crystal structure, anti­bacterial drug, Hirshfeld surface analysis, pairwise inter­action energies

## Abstract

The title compound, C_30_H_33_N_4_O_2_F, can be obtained *via* a two-step synthetic scheme involving 1-benzyl-6-fluoro-4-oxo-7-(piperidin-1-yl)-1,4-di­hydro­quino­line-3-carbo­nitrile as a starting compound that undergoes substitution with hydroxyl­amine and subsequent cyclization with 4-methyl­cyclo­hexane-1-carb­oxy­lic acid. It crystallizes from 2-propanol in the triclinic space group *P*  with a mol­ecule of the title compound and one of 2-propanol in the asymmetric unit. After the mol­ecular structure was clarified using NMR and LC/MS, the mol­ecular and crystalline arrangements were defined with SC-XRD. A Hirshfeld surface analysis was performed for a better understanding of the inter­molecular inter­actions. One strong (O—H⋯O) and three weak [C—H⋯F (intra­molecular) and two C—H⋯O] hydrogen bonds were found. The contributions of short contacts to the Hirshfeld surface were estimated using two-dimensional fingerprint plots showing that O⋯H/H⋯O, C⋯H/H⋯C and C⋯C contacts are the most significant for the title compound and O⋯H for the 2-propanol. The crystal structure appears to have isotropically packed tetra­mers containing two mol­ecules of the title compound and two mol­ecules of 2-propanol as the building unit according to analysis of the distribution of pairwise inter­action energies. A mol­ecular docking study was carried out to evaluate the inter­actions of the title compound with the active centers of macromolecules corresponding to viral targets, namely, anti-hepatitis B activity [HBV, capsid Y132A mutant (VCID 8772) PDB ID: 5E0I] and anti-COVID-19 main protease activity (PDB ID: 6LU7). The data obtained revealed a noticeable affinity towards them that exceeded that of the reference ligands.

## Abstract 2

The synthesis of the potential anti­microbial and anti­viral drug, 5-[1-benzyl-6-fluoro-7-(piperidin-1-yl)-quinolin-4(1*H*)-on-3-yl]-3-(4-methyl­cyclo­hex-1-yl)-1,2,4-oxa­diazole, was proposed. Its mol­ecular and crystal structures were defined and described, whereas the biological activity was predicted with mol­ecular docking.

## 1. Chemical context

One of the promising areas of investigation in the search for new anti­biotic compounds is the synthesis of fluoro­quinolone derivatives. These purely synthetic compounds have been known since 1962 when the first drug from this group, nalidixic acid, was discovered. The scope of their application has changed and substanti­ally broadened from that time since fluorine atoms were included in the 6-position of the quinoline mol­ecule. Today, fluoro­quinolones have positive pharmacokinetic properties, high oral bioavailability, a wide spectrum of action, and good tolerability (Ezelarab *et al.*, 2018 ▸). At present, four generations of fluoro­quinolones exist (Mohammed *et al.*, 2019 ▸), the last of which has found successful use even in the treatment of bacterial pneumonia that developed against the background of COVID-19 (Beović *et al.*, 2020 ▸). Fluoro­quinolones also show their own anti­viral potential (Xu *et al.*, 2019 ▸; Cardoso-Ortiz, *et al.*, 2023 ▸), which opens up prospects for their use in mixed infections. Hence today there is inter­est in new fluoro­quinolones as potential simultaneous anti­bacterial and anti­viral agents. 
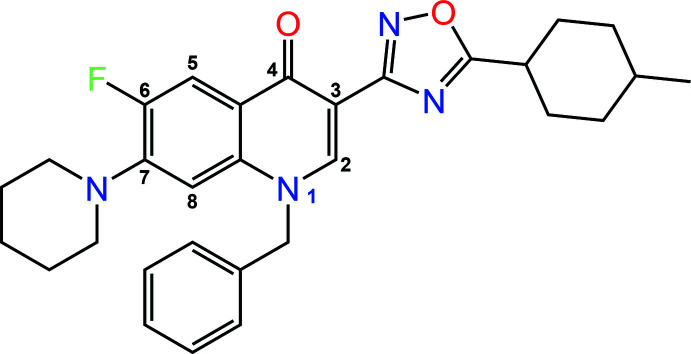


One of the ways to create fluoro­quinolones with combined action is the synthesis of hybrid structures that contain several pharmacophores. The basic fluoro­quinolone mol­ecule can be structurally modified in several directions at once, which significantly expands both the synthetic possibilities and the opportunities for further research into the biological activity of new compounds (Suaifan & Mohammed, 2019 ▸). Structural modification of the bicyclic system of fluoro­quinolones is possible due to the substitution of a nitro­gen atom in position 1, a carboxyl group in position 3, an oxo group in position 4, a fluorine atom in position 6 and by hybridization with heterocyclic nitro­gen fragments in position 7 (see scheme). Thus, hybrids of fluoro­quinolones with derivatives of phenyl­thia­zole, quinazoline, thia­zolidine, thia­diazole, pyrimidine, dithienylethene, and 1,2,4-triazole are widely described in the literature (Suaifan & Mohammed, 2019 ▸; Jia & Zhao, 2021 ▸). Our scientific team has been fruitfully working in this direction (Bylov *et al.*, 1999 ▸; Silin *et al.*, 2004 ▸; Savchenko *et al.*, 2007 ▸; Hryhoriv *et al.*, 2022 ▸; Vaksler *et al.*, 2022 ▸). At the same time, a thorough analysis of literature sources demonstrates that modification of the 3-position of fluoro­quinolones is promising and insufficiently researched (Oniga *et al.*, 2018 ▸). We therefore decided to broaden our investigations of these compounds with studies of 5-[1-benzyl-6-fluoro-7-(piperidin-1-yl)-quin­o­lin-4(1*H*)-on-3-yl]-3-(4-methyl­cyclo­hex-1-yl)-1,2,4-oxa­dia­zole.

## 2. Structural commentary

**Figure 1:**
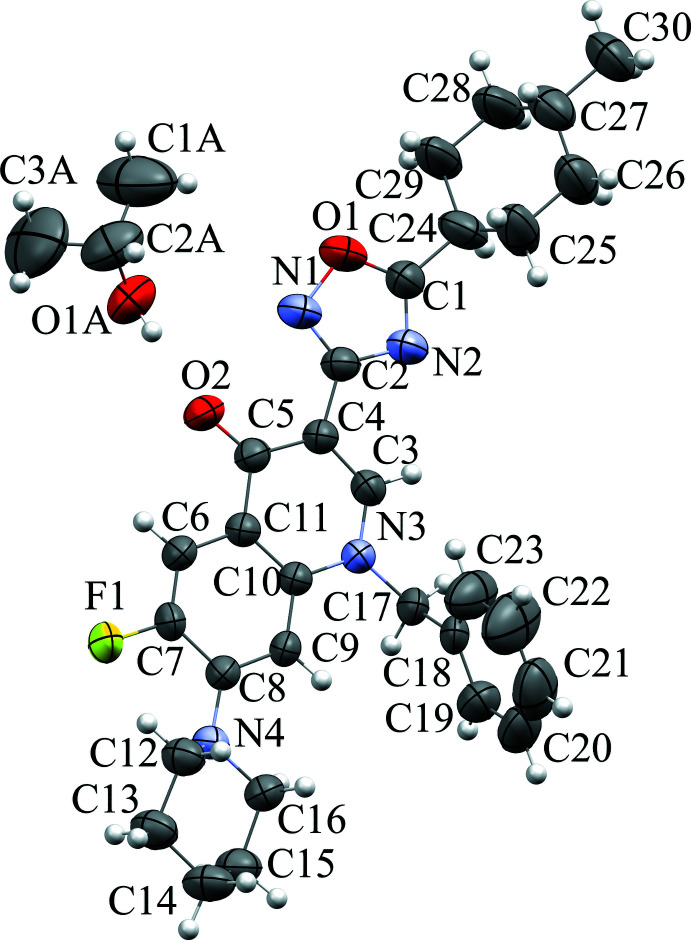
Mol­ecular structure of the title compound. Displacement ellipsoids are shown at the 50% probability level.

The asymmetric unit contains a mol­ecule of 5-[1-benzyl-6-fluoro-7-(piperidin-1-yl)-quinolin-4(1*H*)-on-3-yl]-3-(4-meth­yl­cyclo­hex-1-yl)-1,2,4-oxa­diazole and one of isopropyl alcohol (Fig. 1 ▸). 1,2,4-Oxa­diazole and the quinoline moiety are coplanar [with a dihedral angle between their mean planes of 5.3 (2)° due to π-system conjugation from atom C1 to C8. The piperidine ring is almost unaffected by steric repulsion with other substituents, therefore it is only slightly rotated relative to the bicyclic fragment [the dihedral angle between their mean planes is 28.0 (2)°]. It adopts a chair conformation with puckering parameters (Cremer & Pople, 1975 ▸) *Q* = 0.557 (3) Å, *θ* = 178.0 (3)°, *φ* = 269 (6)°. Atoms N4 and C14 deviate from the mean plane of the other atoms of this ring by 0.652 (2) and −0.696 (3) Å, respectively. Atom N4 has a pyramidal configuration, the sum of bond angles centered at it is 345°. The benzyl substituent is located in the position -*ac* relative to the endocyclic N3–C3 bond [the C3—N3—C17—C18 torsion angle is 103.8 (3)°] and rotated around the N3–C17 bond [N3—C17—C18—C23 = −35.2 (4)°]. The cyclo­hexyl fragment also adopts a chair conformation as well [puckering parameters *Q* = 0.516 (5) Å, *θ* = 177.7 (4)°, *φ* = 331 (10)°] with C24 and C27 deviating from the mean plane through the other ring atoms by 0.590 (3) and −0.622 (4) Å, respectively. It rotates more than the piperidine moiety [the dihedral angle between the mean planes of cyclo­hexane and oxa­diazole derivatives is 46.7 (2)°].

## 3. Supra­molecular features

**Figure 2:**
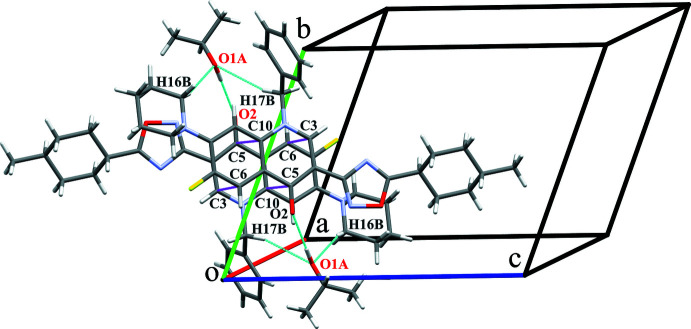
Tetra­meric building unit bonding: hydrogen bonds (in cyan) and short contacts (in magenta).

Regarding the van der Waals radii proposed in Bondi, 1964 ▸ for all the atoms except hydrogen (Rowland & Taylor, 1996 ▸), a very weak non-classical intra­molecular hydrogen bond C12—H12*A*⋯F1 involving the carbon atom from the piperidine moiety (Table 1 ▸) is found together with the three inter­molecular hydrogen bonds of two types. The first type is the strong bond O—H⋯O between the hy­droxy­lic group of isopropyl alcohol and the keto oxygen atom of the main mol­ecule. The isopropyl alcohol mol­ecule is disordered over two positions (*A* and *B*) in a 0.655 (8):0.345 (8) ratio due to a rotation around this hydrogen bond. The weak non-classical C—H⋯O hydrogen bonds involving the oxygen atom of the isopropyl alcohol and methyl­ene groups from the piperidine ring and benzyl moiety belong to the second type. While the bond C16—H16*B*⋯O1*A* exists only for the disordered position *A*, the bond involving the benzyl moiety exists for both disordered positions of the solvent mol­ecule: C17—H17*B*⋯O1*A* and C17—H17*B*⋯O1*B*. In addition, π–π stacking between the quinoline fragments of the original mol­ecule and its symmetry equivalent at −*x*, −*y* + 1, −*z* + 2 [regarding the short contacts C3⋯C6 = 3.379 (4) Å and C5⋯C10 = 3.392 (4) Å] should be mentioned. The mol­ecules of the title compound are bound in pairs with π–π stacking (as the pair of symmetry equivalents *x*, *y*, *z* and −*x*, −*y* + 1, −*z* + 2) and the pairwise inter­actions are complemented by the strong O—H⋯O and weak C—H⋯O hydrogen bonds involving two isopropyl alcohol mol­ecules (symmetry operations: *x* − 1, *y* + 1, *z* and −*x* + 1, −*y*, −*z* + 2). Therefore, the strongly bound tetra­mers (Fig. 2 ▸) consisting of two mol­ecules of the title compound and two mol­ecules of isopropyl alcohol exist in the crystal despite the fact of disorder.

**Figure 3:**
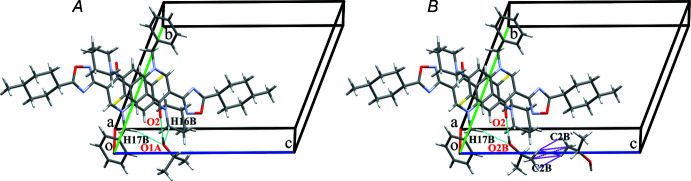
Comparison of hydrogen bonds (in cyan) and short contacts (in magenta) of the 2-propanol mol­ecules in positions *A* and *B*.

Short inter­molecular contacts are also observed. Firstly, the shortening of inter­atomic distances C30⋯F1 [3.130 (6) Å, *x* − 1, *y*, *z* − 1] and H25*B*⋯H25*B*′ [2.260 Å, −*x*, −*y* + 1, −*z* + 1] indicates some of the packing effects influencing the conformation of the *p*-methyl­cyclo­hexyl moiety. Secondly, short contacts exist between the methyl groups of the isopropyl mol­ecules corresponding to neighboring tetra­mers (C2*B*⋯H2*BB*’, 2.56 Å; H2*BB*⋯H2*BB*’, 2.10 Å; C2*B*⋯C2*B*′, 3.26 (3) Å; H2*BA*⋯H2*BB*’, 2.28 Å, symmetry operation: −*x* + 2, −*y*, −*z* + 1). They indicate the existence of repulsion between these isopropyl mol­ecules and so shed light on the reasons for the greater advantage of position *A* (Fig. 3 ▸).

## 4. Hirshfeld surface analysis

**Figure 4:**
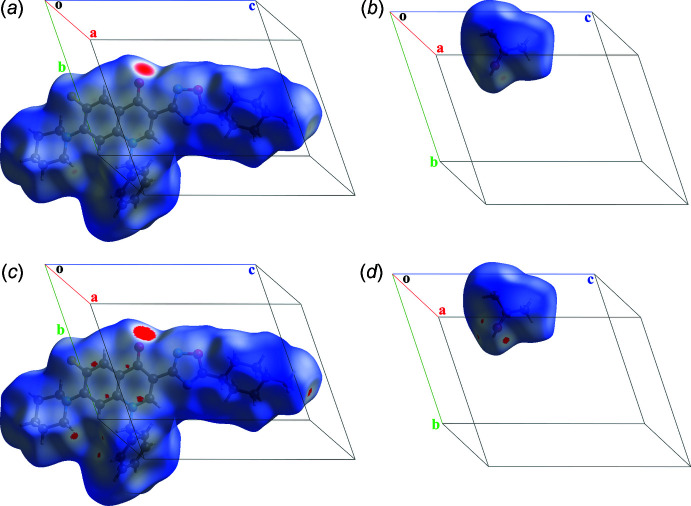
Distribution of the value *d_norm_* onto the Hirshfeld surfaces of the title compound (*a*, *c*) and isopropyl alcohol (*b*, *d*) with default normalization (*a*, *b*) and renormalized (*c*, *d*) for position *A*.

A Hirshfeld surface analysis (Spackman & Byrom, 1997 ▸) was applied to the studied structure. Originally, it was a method that allows the crystal space to be distributed into the regions belonging to different mol­ecules, *i.e.* regions where the specified promolecular electron density exceeds the procrystal density. It was modernized with 2D-fingerprint plots in *CrystalExplorer17* (Spackman *et al.*, 2021 ▸) and can be used for the evaluation of inter­molecular inter­actions. The standard ‘high’ resolution was applied in this study. Four regions with *d*_norm_ significantly lower than the van der Waals contact length (in red) emerge on the surface of the title compound (Fig. 4 ▸ *a*) and two more regions exhibit for the isopropyl alcohol (Fig. 4 ▸ *b*). Indeed, the biggest shortening occurs for the strongest hydrogen bonds: O1*A*—H1*A*⋯O2 or O1*B*—H1*B*⋯O2 in positions *A* and *B*, respectively. The regions where the shortening of *d*_norm_ is less pronounced are associated with the C16—H16*B*⋯O1*A* hydrogen bond and the C30⋯F1 short contact. In addition, with a renormalization of *d*_norm_ from [−0.3991;1.7486] to [−0.0001;1.7486], the appearance of other inter­molecular contacts described above is visible (Fig. 4 ▸ *c*,*d*). As expected, the Hirshfeld surface of the iso­propanol repeats the contacts O—H⋯O and C16—H16*B*⋯O1*A*. Thereby, the appearance of the tetra­mers described above is also confirmed using Hirshfeld surface analysis, especially after renormalization.

**Figure 5:**
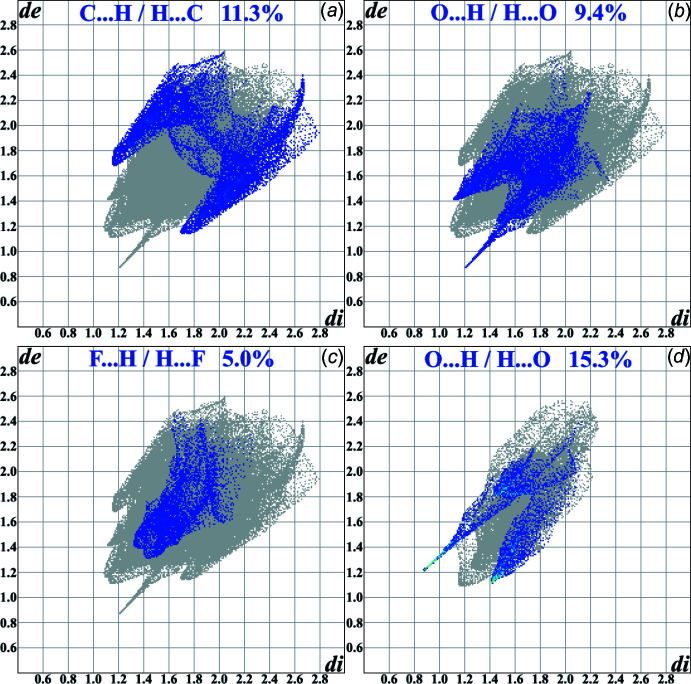
Two-dimensional-fingerprint plots and contributions of the contacts in the title compound [(*a*) C⋯H/H⋯C, (*b*) O⋯H/H⋯O and (*c*) F⋯H / H⋯F] and the isopropyl alcohol mol­ecule (*d*) for position *A*.

**Figure 6:**
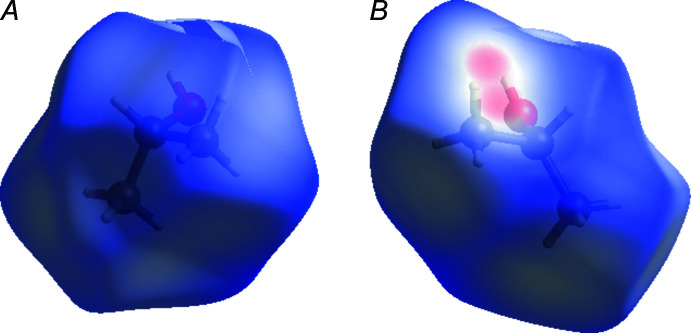
Comparison of the Hirshfeld surfaces of the 2-propanol mol­ecule in positions *A* and *B*.

The 2D-fingerprint plots showed five types of inter­molecular contacts whose contribution into the Hirshfeld surface area for the title compound exceeds 5.0%. They are H⋯H, 61.9%; C⋯H, 11.3%; O⋯H, 9.4%; N⋯H, 5.3%, and F⋯H, 5.0%. However, just three of them relate to areas with the values of the inter­nal and external distances (*d*_i_ and *d*_e_) below the van der Waals radii of the corresponding atoms (Fig. 5 ▸ *a*–*c*). Sharp peaks, whose appearance is usually associated with the formation of inter­molecular inter­actions, are found for the C⋯H and O⋯H contacts, as well as for the ‘less significant’ C⋯C contact (3.9% of the area). They point out the hydrogen bonds and stacking inter­actions in a manner similar to the conventional supra­molecular analysis. Similarly to it, the three contributions exceeding 5.0% of the Hirshfeld surface area are found for isopropyl alcohol: H⋯H, 78.1%; O⋯H, 15.3%, and N⋯H, 5.7%; with just one type below the van der Waals radii of the corresponding atoms: O⋯H (Fig. 5 ▸ *d*). The contributions of inter­molecular contacts do not differ significantly for positions *A* and *B*, except for the appearance of the short contact C2*B*⋯C2*B*′ (Fig. 6 ▸) on the Hirshfeld surface.

## 5. Analysis of the pairwise inter­action energies

The topological analysis allowed us to construct a model of the inter­molecular inter­actions in a crystal. However, this model cannot be confirmed without an assessment of the energetic structure and the contributions of various inter­actions: hydrogen bonds, as obviously strong classical ones, as non-classical ones with a variable and often underestimated strength (Sutor, 1962 ▸; Desiraju, 1996 ▸, 2005 ▸), continuously underrated stacking (Dharmarwardana *et al.*, 2021 ▸; Shishkina *et al.*, 2019 ▸; Zhao & Truhlar, 2008 ▸) and non-specific inter­actions. The procedure proposed by Konovalova *et al.* (2010 ▸) and Shishkin *et al.* (2012 ▸) was applied to define the pairwise inter­action energies of the mol­ecules in crystals in a two-step procedure considering the mol­ecule and the stacking-dimer of mol­ecules as a building unit on a par with the mol­ecule of isopropyl alcohol. The pairs were formed containing the central building unit and its neighboring building units from the first coordination shell. Calculations of the inter­action energies for each pair were performed using the B97 functional (Becke, 1997 ▸; Schmider & Becke, 1998 ▸) with the parameterized three-body (D3) dispersion correction (Grimme *et al.*, 2010 ▸) and Becke–Johnson dumping (Grimme *et al.*, 2011 ▸). The basis set def2-TZVP was used (Weigend & Ahlrichs, 2005 ▸; Weigend, 2006 ▸) and the basis set superposition error (BSSE) correction was implemented according to the Boys–Bernardi counterpoise scheme (Boys & Bernardi, 1970 ▸) in the software package *ORCA 3.0.3* (Neese *et al.*, 2020 ▸). Energy vector diagrams were used for the visualization of the calculated inter­action energies in a standard way (Shishkin *et al.*, 2012 ▸, 2014 ▸). In addition to this, the inter­action energy decomposition was performed using an ‘accurate’ energy model in the program *CrystalExplorer17* for the model with the mol­ecules as building units to clarify the nature of the inter­actions.

**Figure 7:**
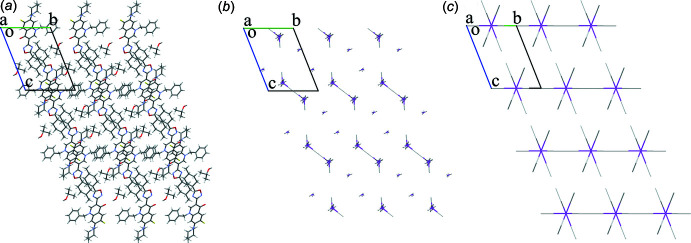
Crystal packing of the mol­ecules (*a*) and energy vector diagrams of the mol­ecules and tetra­mers as building units, (*b*) and (*c*), respectively, for position *A*. Projection in the direction [100].

The inter­actions in the stacking-bonded dimer of the title compound turned out to be two times stronger than any other inter­action of an individual mol­ecule in the crystal structure (∼33.0 kcal mol^−1^). The dispersion is many times superior to the electrostatic and polarization components in it (−28.3 *versus* −9.3 and −3.8 kcal mol^−1^). The other observation is that the aforementioned tetra­mers contain the sole inter­action of the title mol­ecules in the crystal that has the electrostatic component higher than the dispersive one (−7.7 and −8.5 kcal mol^−1^ in total for positions *A* and *B*, respectively, −2.6 *versus* −5.4 and −2.2 kcal mol^−1^ in components in position *A*). It is also the strongest inter­action for the iso­propanol mol­ecule (Table 2 ▸). The other inter­action for which the hydrogen bonding is at least comparable to the dispersion component is also included in the tetra­mer with C—H⋯O bonding (−4.4 and −5.9 kcal mol^−1^ in total for positions *A* and *B*, respectively, −5.8 *versus* −2.8 and −1.1 kcal mol^−1^ in the components in position *A*). Since hydrogen bonding is a directed and thus more rigid inter­action than the non-specific inter­actions like dispersion, the tetra­mers including two mol­ecules of the title compound and two mol­ecules of isopropyl alcohol can be distinguished as a basic structural motif (Fig. 7 ▸). It can be added that the last inter­action with the hydrogen bonding comparable to the dispersion component within the structure appears in between the tetra­mers with the symmetry equivalent −*x* + 1, −*y* + 1, −*z* + 2 of the title compound (−11.8 kcal mol^−1^ in total; −8.5 *versus* −4.3 and −2.2 kcal mol^−1^ in the components). However, any short contacts or hydrogen bonds are not observable in this pair of mol­ecules and the inter­actions of tetra­mers are completely isotropic according to the energies (Table 3 ▸). So, the influence of hydrogen bonding seems limited solely to the additional connection of the stacking-bonded dimer. The second strongest inter­action of the title compound has a dispersive nature. It is the inter­action of the *p*-methyl­cyclo­hexyl moieties of the original mol­ecule and the symmetry equivalent −*x*, −*y* + 1, −*z* + 1 (−15.0 kcal mol^−1^ in total; −14.6 *versus* −1.7 and −0.9 kcal mol^−1^ in the components). At that, the influence of the short contact C30⋯F1 is negligible, because the inter­action energy in the dimer including the symmetry equivalent *x* − 1, *y*, *z* − 1 of the title compound is very small (−1.8 kcal mol^−1^ in total). Since the electrostatic inter­actions are weak around the *p*-methyl­cyclo­hexyl moiety, it is possible to notice that the rotation of the corresponding group is affected mostly by the dispersive inter­actions among the inter­molecular ones. The iso­propanol exhibits strong hydrogen bonding within the tetra­mers, but its mol­ecules endure just minor inter­actions with the neighbors not connected by hydrogen bonds (Table 2 ▸). The last observation is that the disorder and the subsequent shortening of the distance between the iso­propanol mol­ecules in position *B* (the short contact C2*B*⋯C2*B*′) significantly affects the inter­molecular inter­actions in the corresponding pairs of iso­propanol mol­ecules (−1.4 *versus* 1.5 kcal mol^−1^ in positions *A* and *B*, respectively). This leads to a loss in the total inter­action energy from −23.4 to −20.6 kcal mol^−1^ in position *B*, making it less energetically favorable for the isopropyl alcohol because of the steric repulsion (lower dispersive inter­action) of the methyl moieties.

## 6. Mol­ecular docking

To estimate the potential biological properties and the possible inter­actions of the title compound with the active centers of target viral macromolecules, we conducted a mol­ecular docking study. Two targets from the Protein Data Bank (PDB) were utilized for this purpose. The first one is the capsid of the Hepatitis B virus (HBV capsid Y132A mutant VCID 8772, PDB ID: 5E0I; Klumpp, *et al.*, 2015 ▸). The second is COVID-19 main protease PDB ID: 6LU7 (Jin *et al.*, 2020 ▸).

The crystal structure of the HBV capsid Y132A contains 157 amino acid residues; the mol­ecular weight is 109.09 kDa. Methyl 4-(2-bromo-4-fluoro­phen­yl)-6-(morpholin-4-ylmeth­yl)-2-(1,3-thia­zol-2-yl)pyrimidine-5-carboxyl­ate was used as a reference ligand. For 5E0I there are six protein chains designated as *A*, *B*, *C*, *D*, *E*, and *F*. According to our computations, the residual mean squared deviation between experimental data from X-ray diffraction analysis and the docking-generated position is around 1 Å, which is even better than the X-ray resolution reported for the structures (1.95 and 2.16 Å, respectively). Hence, for the docking procedure, we can use any of the above-mentioned chains. We used chain *A* in the actual calculations.

The crystal structure of the COVID-19 main protease contains 306 amino acid residues; the mol­ecular weight is 34.51 kDa. *N*-[(5-Methyl­isoxazol-3-yl)carbon­yl]alanyl-l-valyl-*N*1-(1*R*,2Z)-4-(benz­yloxy)-4-oxo-1-{[(3*R*)-2-oxopyrrolidin-3-yl]meth­yl}but-2-en­yl)-l-leucinamide was used as a reference ligand.

These target macromolecules had previously been utilized for similar research, and therefore we proceeded with them both to obtain the docking results, and determine whether we could enhance the anti­viral properties that were observed for some fluoro­quinolones that had been hybridized with heterocycles.

For the graphical analysis, the free software packages *Jmol* (Jmol, 2022 ▸) and *PyMol* (DeLano, 2002 ▸) were used. The virtual screening, pharmacophore investigation, and mol­ecular docking procedures, with the subsequent analysis of their data, were performed using the *LigandScout 4.4* software complex (Wolber & Langer, 2005 ▸). For the calculations of standard mol­ecular QSAR parameters, the popular resource *SwissADME* from the Swiss Institute of Bioinformatics (Daina *et al.*, 2017 ▸) was utilized.

According to the results of docking studies, it was found that the tested mol­ecule has a significant affinity to both targets (Table 4 ▸). This is evidenced by the values of the scoring functions and the free binding energy in comparison to the values of the described reference ligands. Among the QSAR properties obtained from *SwissADME*, we included only the most important, namely MlogP (calculated by using the Moriguchi approach), LogS (by ESOL) and topological polar surface area (TPSA) (Daina, *et al.*, 2017 ▸). These parameters are important characteristics of the transport properties of a drug through membranes. While the LogS and TPSA parameters correspond to the drug likeliness criterion, the lipophilicity for the title compound is noticeably larger. However, this difficulty can potentially be eliminated by some structural chemical modifications, for example, by incorporating appropriate substituents.

**Figure 8:**
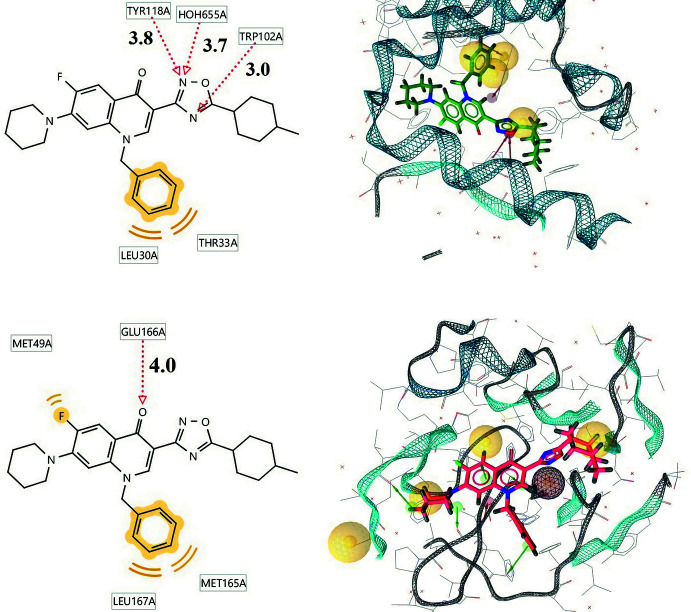
Inter­actions (on the left) and configuration of the title compound (on the right) within the active centers of target viral macromolecules (5E0I – at the top, 6LU7 – at the bottom).

An analysis of the geometric location in the active sites of the selected targets showed that the formation of complexes is facilitated by hydrogen bonds (shown with dotted red arrows) and hydro­phobic (van der Waals) inter­molecular inter­actions (designated in yellow) (Fig. 8 ▸). The *D*⋯*A* distances involved in hydrogen bonds (in Å) are presented in the left part of this figure. It can be seen that the obtained lengths are quite large, but fall within the typical range for hydrogen bonds (2.5–4.0 Å).

Therefore, the investigated compound is promising for further *in vitro* research of both the anti­microbial and anti­viral activity.

## 7. Synthesis and crystallization

The starting reagents are commercially available and, as well as solvents, were purchased from Sigma Aldrich and were used without further purification.

The initial substance (compound **1** in the reaction scheme), 1-benzyl-6-fluoro-4-oxo-7-(piperidin-1-yl)-1,4-di­hydro­quin­o­line-3-carbo­nitrile, was synthesized *via* N-alkyl­ation of 6,7-di­fluoro­quinolin-4-one-3-nitrile, followed by amination of the resultant inter­mediates with piperidine according to the procedures described in Spiridonova *et al.* (2011 ▸). This compound is convenient for further cyclization reactions to obtain various heterocyclic-substituted derivatives. Furthermore, this method is promising for the creation of new substances with anti­microbial activity.

Compound **1** (1 mmol) and *N*,*N*′-carbonyl­diimidazole (1.1 mmol) were dissolved in *N*,*N*-di­methyl­formamide and stirred at 373 K for 20 minutes. After that hydroxyl­amine (1.1 mmol) was added and heating was maintained for 6 h. The mixture was cooled to room temperature, then water was added, and the obtained precipitate was filtered and recrystallized from an iso­propanol–DMF mixture. Then 1 mmol of the obtained compound (**2**), *N*,*N*′-carbonyl­diimidazole (1.1 mmol) (compound **3**) and 4-methyl­cyclo­hexane-1-carb­oxy­lic acid (1 mmol) (compound **4**) were dissolved in *N*,*N*-di­methyl­formamide (80 mL). The mixture was stirred at 333 K for 1 h. The mixture was then cooled to room temperature and the obtained precipitate was filtered, washed with ethanol and recrystallized from an ethanol–DMF mixture. 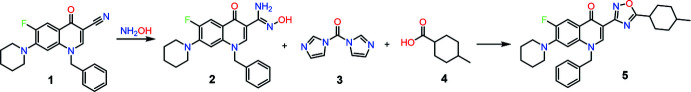


Further crystallization by slow evaporation of a solution in iso­propanol was carried out to provide single block-like colorless crystals suitable for X-ray diffraction analysis (m.p. 476–477 K).

## 8. NMR and LC/MS characterization

The NMR spectra were recorded on a Varian MR-400 spectrometer with standard pulse sequences operating at 400 MHz for ^1^H NMR and 101 MHz for ^13^C NMR. For the NMR spectra, DMSO-*d*_6_ was used as a solvent. Chemical shift values are referenced to residual protons (δ 2.49 ppm) and carbons (δ 39.6 ppm) of the solvent as an inter­nal standard.

LC/MS spectra were recorded on an ELSD Alltech 3300 liquid chromatograph equipped with a UV detector (λ_max_ = 254 nm), API-150EX mass-spectrometer and using a Zorbax SB-C18 column, Phenomenex (100 × 4 mm) Rapid Resolution HT Cartridge 4.6×30mm, 1.8-Micron. Elution started with a 0.1 *M* solution of HCOOH in water and ended with a 0.1 *M* solution of HCOOH in aceto­nitrile using a linear gradient at a flow rate of 0.15 ml min^−1^ and an analysis cycle time of 25 min.

Characteristics of title mol­ecule: LC/MS: [MH]^+^ = 501.26. ^1^H NMR (400 MHz, DMSO-*d*_6_) δ 9.08 (*s*, 1H, H-2), 8.06 (*d*, *J* = 12.0 Hz, 1H, H-5), 7.35–7.23 (*m*, 5H, **Ph**-CH_2_), 6.31 (*d*, *J* = 4.4 Hz, 1H, H-8), 5.43 (*s*, 2H, Ph-**CH_2_**), 3.27–3.20 (*m*, 4H, piperidine H-2), 3.13 (*p*, *J* = 6.5 Hz, 1H, cyclo­hexane H-1), cyclo­aliphatics: [2.24–2.14 (*m*, 2H), 1.98–1.88 (*m*, 2H), 1.73–1.45 (*m*, 9H), 1.35–1.23 (*m*, 2H)], 0.91 (*d*, *J* = 5.9 Hz, 3H, CH_3_). ^13^C NMR (101 MHz, DMSO-*d*_6_) δ 181.35, 181.33, 178.80, 165.60, 151.45, 149.00, 146.21, 144.87, 144.75, 138.83, 138.80, 137.36, 129.16, 128.13, 127.52, 122.73, 122.66, 113.97, 113.75, 104.96, 104.91, 104.27, 54.03, 50.90, 50.86, 33.49, 33.36, 30.48, 27.72, 25.90, 24.07, 20.97.

## 9. Database survey

A search of the Cambridge Structural Database (Version 5.42, update of November 2020; Groom *et al.*, 2016 ▸) did not show any closely related structures to the 5-[1-benzyl-6-fluoro-7-(piperidin-1-yl)-quinolin-4(1*H*)-on-3-yl]-3-(4-methyl­cyclo­hex-1-yl)-1,2,4-oxa­diazole. The compound with the closest structure is *N*-benzyl-1-butyl-6-fluoro-4-oxo-7-(piperidin-1-yl)-1,4-di­hydro­quinoline-3-carboxamide (Hiltensperger *et al.*, 2012 ▸), which belongs, however, to another class of anti­parasitic fluoro­quinolone derivatives.

## 10. Refinement

Crystal data, data collection and structure refinement details are summarized in Table 5 ▸. All hydrogen atoms were refined using a riding model with *U*_iso_ = *nU*_eq_ of the carrier atom (*n* = 1.5 for methyl and hydroxyl groups and *n* = 1.2 for other hydrogen atoms). During the refinement, the distances between the atoms of the disordered isopropyl alcohol were restrained to the following values: 1.524 Å for bonds C1*A*—C2*A*, C1*B*—C2*B*, C1*A*—C3*A*, C1*B*—C3*B* with an estimated standard deviation of 0.015 Å (according to Dunitz & Bürgi, 1994 ▸) and 1.432 Å for bonds O1*A*—C1*A*/O1*B*—C1*B* with an estimated standard deviation of 0.011 Å. The atoms of each disordered position of the isopropyl alcohol were restrained to have the same *U*_ij_ components with an estimated standard deviation of 0.01 Å^2^ (0.02 Å^2^ for terminal atoms). They were subject to a ‘rigid bond’ restraint as well with an estimated standard deviation of 0.0025 Å^2^.

## Supplementary Material

Crystal structure: contains datablock(s) I. DOI: 10.1107/S2056989023001305/dx2050sup1.cif

Structure factors: contains datablock(s) I. DOI: 10.1107/S2056989023001305/dx2050Isup2.hkl

Supporting information file. DOI: 10.1107/S2056989023001305/dx2050Isup3.cml

CCDC reference: 2241448

Additional supporting information:  crystallographic information; 3D view; checkCIF report

## Figures and Tables

**Table 1:** Hydrogen-bond geometry (Å, °)

*D*—H⋯*A*	*D*—H	H⋯*A*	*D*⋯*A*	*D*—H⋯*A*
C12—H12*A*⋯F1	0.97	2.19	2.873 (4)	126
C16—H16*B*⋯O1*A* ^i^	0.97	2.53	3.441 (9)	155
C17—H17*B*⋯O1*A* ^i^	0.97	2.59	3.504 (9)	157
C17—H17*B*⋯O1*B* ^i^	0.97	2.55	3.491 (17)	162
O1*A*—H1*A*⋯O2^ii^	0.82	2.09	2.906 (9)	180
O1*B*—H1*B*⋯O2^ii^	0.82	1.84	2.662 (19)	180

Symmetry codes: (i) 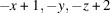 ; (ii) 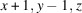 .

**Table 2:** Symmetry codes, binding types and inter­action energies (kcal mol^−1^) of the building units (BU) (single mol­ecules) with neighbors

Pair of BU	Central BU	Neighboring BU and corresponding symmetry operation	*E*_int_	Inter­action
Position *A*				
1	TC	*x* − 1, *y*, *z* − 1; TC	−1.8	C30⋯F1
2	TC	*x* − 1, *y* + 1, *z* − 1; TC	−3.9	Non-specific
3	TC	-*x* + 2, −*y*, −*z* + 2; IP	−1.6	Non-specific
4	TC	*x*, *y* − 1, *z*; TC	−2.6	Non-specific
5	TC	*x*, *y*, *z* + 1; IP	−1.1	Non-specific
6	TC	-*x* + 1, −*y* + 1, −*z* + 2; TC	−11.8	Non-specific
7	TC	-*x* + 1, −*y* + 1, −*z* + 1; IP	−2.8	Non-specific
8	TC	-*x* + 1, −*y*, −*z* + 3; TC	−1.8	Non-specific
9	TC	-*x* + 1, −*y*, −*z* + 2; TC	−9.2	Non-specific
10	TC	-*x* + 1, −*y*, −*z* + 2; IP	−4.4	C16—H16*B*⋯O1*A*, C17—H17*B*⋯O1*A*
11	TC	*x*, *y* + 1, *z*; TC	−2.6	Non-specific
12	TC	*x* − 1, *y* + 1, *z*; IP	−7.7	O1*A*—H1*A*⋯O2
13	TC	*x* + 1, *y* − 1, *z* + 1; TC	−3.9	Non-specific
14	TC	-*x*, −*y* + 1, −*z* + 2; TC	−33.0	C3⋯C6, C5⋯C10
15	TC	*x* − 1, *y*, *z*; IP	−3.6	Non-specific
16	TC	-*x*, −*y* + 1, −*z* + 1; TC	−15.0	Non-specific
17	TC	-*x*, −*y* + 1, −*z* + 1; IP	−0.8	Non-specific
18	TC	*x* + 1, *y*, *z* + 1; TC	−1.8	C30⋯F1
19	TC	-*x*, −*y*, −*z* + 2; TC	−8.3	Non-specific
20	TC	-*x* − 1, −*y* + 1, −*z* + 1; TC	−4.2	Non-specific
21	IP	*x* + 1, *y*, *z*; TC	−3.6	Non-specific
22	IP	-*x* + 2, −*y*, −*z* + 2; TC	−1.6	Non-specific
23	IP	*x* + 1, *y* − 1, *z*; TC	−7.7	O1*A*—H1*A*⋯O2
24	IP	-*x* + 2, −*y*, −*z* + 1; IP	−1.4	Non-specific
25	IP	-*x* + 1, −*y* + 1, −*z* + 1; TC	−2.8	Non-specific
26	IP	*x*, *y*, *z* − 1; TC	−1.1	Non-specific
27	IP	–*x* + 1, −*y*, −*z* + 2; TC	−4.4	C16—H16*B*⋯O1*A*, C17—H17*B*⋯O1*A*
28	IP	-*x*, −*y* + 1, −*z* + 1; TC	−0.8	Non-specific
				
Position *B*				
1	TC	*x* − 1, *y*, *z* − 1; TC	−1.8	C30⋯F1
2	TC	*x* − 1, *y* + 1, *z* − 1; TC	−3.9	Non-specific
3	TC	-*x* + 2, −*y*, −*z* + 2; IP	−1.3	Non-specific
4	TC	*x*, *y* − 1, *z*; TC	−2.6	Non-specific
5	TC	*x*, *y*, *z* + 1; IP	−0.9	Non-specific
6	TC	-*x* + 1, −*y* + 1, −*z* + 2; TC	−11.8	Non-specific
7	TC	-*x* + 1, −*y* + 1, −*z* + 1; IP	−1.9	Non-specific
8	TC	-*x* + 1, −*y*, −*z* + 3; TC	−1.8	Non-specific
9	TC	-*x* + 1, −*y*, −*z* + 2; TC	−9.2	Non-specific
10	TC	-*x* + 1, −*y*, −*z* + 2; IP	−5.9	C16—H16*B*⋯O1*B*, C17—H17*B*⋯O1*B*
11	TC	*x*, *y* + 1, *z*; TC	−2.6	Non-specific
12	TC	*x* − 1, *y* + 1, *z*; IP	−8.5	O1*B*—H1*B*⋯O2
13	TC	*x* + 1, *y* − 1, *z* + 1; TC	−3.9	Non-specific
14	TC	-*x*, −*y* + 1, −*z* + 2; TC	−33.0	C3⋯C6, C5⋯C10
15	TC	*x* − 1, *y*, *z*; IP	−2.4	Non-specific
16	TC	-*x*, −*y* + 1, −*z* + 1; TC	−15.0	Non-specific
17	TC	-*x*, −*y* + 1, −*z* + 1; IP	−1.1	Non-specific
18	TC	*x* + 1, *y*, *z* + 1; TC	−1.8	C30⋯F1
19	TC	-*x*, −*y*, −*z* + 2; TC	−8.3	Non-specific
20	TC	-*x* − 1, −*y* + 1, −*z* + 1; TC	−4.2	Non-specific
21	IP	*x* + 1, *y*, *z*; TC	−2.4	Non-specific
22	IP	-*x* + 2, −*y*, −*z* + 2; TC	−1.3	Non-specific
23	IP	*x* + 1, *y* − 1, *z*; TC	−8.5	O1*B*—H1*B*⋯O2
24	IP	-*x* + 2, −*y*, −*z* + 1; IP	1.5	Non-specific
25	IP	-*x* + 1, −*y* + 1, −*z* + 1; TC	−1.9	Non-specific
26	IP	*x*, *y*, *z* − 1; TC	−0.9	Non-specific
27	IP	-*x* + 1, −*y*, −*z* + 2; TC	−5.9	C16—H16*B*⋯O1*B*, C17—H17*B*⋯O1*B*
28	IP	-*x*, −*y* + 1, −*z* + 1; TC	−1.1	Non-specific

**Table 3:** Symmetry codes, binding types and inter­action energies of the building units (BU) (kcal mol^−1^) (tetra­mers) with neighbors

Pair of BU	Symmetry operation for neighboring BU	*E*_int_
Position *A*		
1	*x* − 1, *y*, *z* − 1	−7.9
2	*x* − 1, *y*, *z*	−13.9
3	*x* − 1, *y* + 1, *z* − 1	−13.0
4	*x* − 1, *y* + 1, *z*	−9.7
5	*x*, *y* − 1, *z*	−19.5
6	*x*, *y* − 1, *z* + 1	−7.5
7	*x*, *y*, *z* − 1	−15.7
8	*x*, *y*, *z* + 1	−15.7
9	*x*, *y* + 1, *z* − 1	−7.5
10	*x*, *y* + 1, *z*	−19.5
11	*x* + 1, *y* − 1, *z*	−9.7
12	*x* + 1, *y* − 1, *z* + 1	−13.0
13	*x* + 1, *y*, *z*	−13.9
14	*x* + 1, *y*, *z* + 1	−7.9
		
Position *B*		
1	*x* − 1, *y*, *z* − 1	−7.9
2	*x* − 1, *y*, *z*	−13.9
3	*x* − 1, *y* + 1, *z* − 1	−13.2
4	*x* − 1, *y* + 1, *z*	−9.7
5	*x*, *y* − 1, *z*	−17.0
6	*x*, *y* − 1, *z* + 1	−2.9
7	*x*, *y*, *z* − 1	−15.8
8	*x*, *y*, *z* + 1	−15.8
9	*x*, *y* + 1, *z* − 1	−2.9
10	*x*, *y* + 1, *z*	−17.0
11	*x* + 1, *y* − 1, *z*	−9.7
12	*x* + 1, *y* − 1, *z* + 1	−13.2
13	*x* + 1, *y*, *z*	−13.9
14	*x* + 1, *y*, *z* + 1	−7.9

**Table 4:** Basic characteristics of mol­ecular docking

Compounds	BE (kcal mol^−1^)	Binding affinity score	MLogP	LogS (ESOL)	TPSA (Å^2^)
Ligand + 5E0I	−6.9	−17.34	1.76	−4.65	105.68
Title compound + 5E0I	−8.8	−18.03	4.50	−7.19	64.16
Ligand + 6LU7	−7.6	−21.86	0.38	−4.89	197.83
Title compound + 6LU7	−9.1	−19.61	4.50	−7.19	64.16

**Table 5:** Experimental details

Crystal data	
Chemical formula	C_30_H_33_FN_4_O_2_·C_3_H_8_O
*M*_r_	560.70
Crystal system, space group	Triclinic, *P* 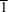
Temperature (K)	296
*a*, *b*, *c* (Å)	9.9499 (6), 11.2976 (9), 14.9913 (12)
α, β, γ (°)	68.650 (8), 89.473 (6), 78.976 (6)
*V* (Å^3^)	1537.2 (2)
*Z*	2
Radiation type	Mo *K*α
μ (mm^−1^)	0.08
Crystal size (mm)	0.18 × 0.14 × 0.1

Data collection	
Diffractometer	Xcalibur, Atlas
Absorption correction	Multi-scan (*CrysAlis PRO*; Rigaku OD, 2021 ▸)
*T*_min_, *T*_max_	0.986, 1.000
No. of measured, independent and observed [*I* > 2σ(*I*)] reflections	14562, 5407, 2707
*R*_int_	0.043
(sin θ/λ)_max_ (Å^−1^)	0.595

Refinement	
*R*[*F* ^2^ > 2σ(*F* ^2^)], *wR*(*F* ^2^), *S*	0.066, 0.189, 0.97
No. of reflections	5407
No. of parameters	412
No. of restraints	54
H-atom treatment	H-atom parameters constrained
Δρ_max_, Δρ_min_ (e Å^−3^)	0.30, −0.24

Computer programs: *CrysAlis PRO* (Rigaku OD, 2021 ▸), *SHELXT2014/5* (Sheldrick, 2015*a* ▸), *SHELXL2019/2* (Sheldrick, 2015*b* ▸) and *OLEX2* (Dolomanov *et al.*, 2009 ▸).

## supplementary crystallographic information

### Crystal data

**Table:**

C_30_H_33_FN_4_O_2_·C_3_H_8_O	*F*(000) = 600
*M**_r_* = 560.70	*D*_x_ = 1.211 Mg m^−^^3^
Triclinic, *P*1	Melting point: 476.15 K
*a* = 9.9499 (6) Å	Mo *K*α radiation, λ = 0.71073 Å
*b* = 11.2976 (9) Å	Cell parameters from 2589 reflections
*c* = 14.9913 (12) Å	θ = 4.0–28.2°
α = 68.650 (8)°	µ = 0.08 mm^−^^1^
β = 89.473 (6)°	*T* = 296 K
γ = 78.976 (6)°	Block, yellow
*V* = 1537.2 (2) Å^3^	0.18 × 0.14 × 0.1 mm
*Z* = 2	

### Data collection

**Table:**

Xcalibur, Atlas diffractometer	5407 independent reflections
Radiation source: fine-focus sealed X-ray tube, Enhance (Mo) X-ray Source	2707 reflections with *I* > 2σ(*I*)
Graphite monochromator	*R*_int_ = 0.043
Detector resolution: 10.3779 pixels mm^-1^	θ_max_ = 25.0°, θ_min_ = 3.4°
ω scans	*h* = −11→11
Absorption correction: multi-scan (CrysAlisPro; Rigaku OD, 2021)	*k* = −12→13
*T*_min_ = 0.986, *T*_max_ = 1.000	*l* = −16→17
14562 measured reflections	

### Refinement

**Table:**

Refinement on *F*^2^	Primary atom site location: structure-invariant direct methods
Least-squares matrix: full	Hydrogen site location: mixed
*R*[*F*^2^ > 2σ(*F*^2^)] = 0.066	H-atom parameters constrained
*wR*(*F*^2^) = 0.189	*w* = 1/[σ^2^(*F*_o_^2^) + (0.0881*P*)^2^] where *P* = (*F*_o_^2^ + 2*F*_c_^2^)/3
*S* = 0.97	(Δ/σ)_max_ < 0.001
5407 reflections	Δρ_max_ = 0.30 e Å^−^^3^
412 parameters	Δρ_min_ = −0.24 e Å^−^^3^
54 restraints	

### Special details

**Table:**

Geometry. All esds (except the esd in the dihedral angle between two l.s. planes) are estimated using the full covariance matrix. The cell esds are taken into account individually in the estimation of esds in distances, angles and torsion angles; correlations between esds in cell parameters are only used when they are defined by crystal symmetry. An approximate (isotropic) treatment of cell esds is used for estimating esds involving l.s. planes.

### Fractional atomic coordinates and isotropic or equivalent isotropic displacement parameters (Å^2^)

**Table:**

	*x*	*y*	*z*	*U*_iso_*/*U*_eq_	Occ. (<1)
F1	0.40408 (18)	0.53219 (16)	1.12186 (12)	0.0810 (6)	
O1	−0.1510 (2)	0.7148 (3)	0.59426 (15)	0.0918 (7)	
O2	0.10050 (19)	0.6960 (2)	0.82423 (13)	0.0688 (6)	
N1	−0.0680 (3)	0.6992 (3)	0.67646 (18)	0.0839 (8)	
N2	−0.1356 (3)	0.5139 (3)	0.69218 (17)	0.0729 (7)	
N3	0.0757 (2)	0.3148 (2)	0.95995 (15)	0.0535 (6)	
N4	0.4118 (2)	0.2672 (2)	1.21202 (14)	0.0543 (6)	
C1	−0.1860 (3)	0.6009 (4)	0.6103 (2)	0.0727 (9)	
C2	−0.0628 (3)	0.5791 (3)	0.73033 (19)	0.0595 (8)	
C3	0.0080 (3)	0.3883 (3)	0.87466 (19)	0.0564 (7)	
H3	−0.047481	0.350041	0.848437	0.068*	
C4	0.0143 (3)	0.5143 (3)	0.82355 (18)	0.0524 (7)	
C5	0.0951 (3)	0.5791 (3)	0.86249 (18)	0.0528 (7)	
C6	0.2576 (3)	0.5490 (3)	0.99624 (18)	0.0548 (7)	
H6	0.263435	0.635774	0.967296	0.066*	
C7	0.3312 (3)	0.4740 (3)	1.07963 (19)	0.0554 (7)	
C8	0.3322 (3)	0.3400 (3)	1.12679 (18)	0.0510 (7)	
C9	0.2445 (3)	0.2905 (3)	1.08445 (17)	0.0495 (7)	
H9	0.239041	0.203628	1.113368	0.059*	
C10	0.1645 (2)	0.3672 (3)	0.99992 (17)	0.0479 (7)	
C11	0.1729 (3)	0.4969 (3)	0.95330 (17)	0.0495 (7)	
C12	0.5588 (3)	0.2719 (3)	1.21148 (19)	0.0655 (8)	
H12A	0.569509	0.360773	1.178841	0.079*	
H12B	0.605173	0.220547	1.176408	0.079*	
C13	0.6240 (3)	0.2206 (3)	1.3121 (2)	0.0767 (10)	
H13A	0.720944	0.222963	1.309746	0.092*	
H13B	0.581915	0.275375	1.345936	0.092*	
C14	0.6068 (3)	0.0834 (3)	1.3656 (2)	0.0829 (11)	
H14A	0.642767	0.054073	1.431599	0.100*	
H14B	0.657452	0.026422	1.336142	0.100*	
C15	0.4562 (3)	0.0782 (3)	1.3629 (2)	0.0732 (9)	
H15A	0.446362	−0.011460	1.392007	0.088*	
H15B	0.408760	0.125075	1.400558	0.088*	
C16	0.3905 (3)	0.1353 (3)	1.26275 (19)	0.0602 (8)	
H16A	0.428692	0.081034	1.227556	0.072*	
H16B	0.292786	0.136451	1.265326	0.072*	
C17	0.0544 (3)	0.1821 (3)	1.0101 (2)	0.0596 (8)	
H17A	−0.029777	0.173344	0.983203	0.072*	
H17B	0.042178	0.168847	1.077120	0.072*	
C18	0.1692 (3)	0.0775 (3)	1.0047 (2)	0.0613 (8)	
C19	0.2007 (4)	−0.0381 (3)	1.0801 (3)	0.0839 (10)	
H19	0.153116	−0.049765	1.135303	0.101*	
C20	0.3014 (5)	−0.1382 (4)	1.0764 (4)	0.1067 (13)	
H20	0.319731	−0.216775	1.128258	0.128*	
C21	0.3726 (5)	−0.1226 (5)	0.9984 (5)	0.1236 (16)	
H21	0.441061	−0.190020	0.996214	0.148*	
C22	0.3450 (6)	−0.0083 (5)	0.9226 (4)	0.150 (2)	
H22	0.395091	0.002815	0.868434	0.180*	
C23	0.2423 (5)	0.0921 (4)	0.9254 (3)	0.1159 (15)	
H23	0.223190	0.169991	0.872837	0.139*	
C24	−0.2713 (3)	0.5875 (4)	0.5338 (2)	0.0853 (11)	
H24	−0.344876	0.544481	0.565601	0.102*	
C25	−0.1882 (4)	0.5005 (4)	0.4899 (3)	0.1171 (15)	
H25A	−0.157137	0.415446	0.539389	0.141*	
H25B	−0.107661	0.534888	0.464956	0.141*	
C26	−0.2665 (4)	0.4864 (4)	0.4101 (3)	0.1127 (14)	
H26A	−0.205076	0.434701	0.381433	0.135*	
H26B	−0.339078	0.440211	0.436824	0.135*	
C27	−0.3268 (4)	0.6112 (4)	0.3353 (2)	0.0948 (12)	
H27	−0.250641	0.653171	0.307528	0.114*	
C28	−0.4127 (5)	0.6966 (4)	0.3779 (3)	0.1274 (16)	
H28A	−0.492846	0.660744	0.401895	0.153*	
H28B	−0.444442	0.781228	0.328072	0.153*	
C29	−0.3383 (5)	0.7134 (4)	0.4595 (3)	0.1212 (15)	
H29A	−0.269221	0.764582	0.433456	0.145*	
H29B	−0.403422	0.760878	0.489193	0.145*	
C30	−0.4034 (4)	0.5969 (5)	0.2541 (3)	0.1172 (14)	
H30A	−0.482705	0.561190	0.277632	0.176*	
H30B	−0.344326	0.539912	0.229418	0.176*	
H30C	−0.431828	0.680354	0.203884	0.176*	
O1A	0.9230 (8)	−0.0496 (7)	0.7385 (6)	0.090 (2)	0.655 (8)
H1A	0.973132	−0.121327	0.762874	0.135*	0.655 (8)
C1A	0.9840 (10)	0.0330 (8)	0.6585 (6)	0.119 (3)	0.655 (8)
H1AA	1.064792	−0.015019	0.639707	0.143*	0.655 (8)
C2A	0.8698 (11)	0.0861 (12)	0.5824 (7)	0.189 (5)	0.655 (8)
H2AA	0.845004	0.016382	0.568347	0.283*	0.655 (8)
H2AB	0.898808	0.146171	0.525414	0.283*	0.655 (8)
H2AC	0.791923	0.130018	0.604323	0.283*	0.655 (8)
C3A	1.0152 (10)	0.1391 (9)	0.6815 (7)	0.195 (5)	0.655 (8)
H3AA	0.936961	0.176312	0.707382	0.292*	0.655 (8)
H3AB	1.037392	0.204036	0.624393	0.292*	0.655 (8)
H3AC	1.091995	0.107152	0.728049	0.292*	0.655 (8)
O1B	0.957 (2)	−0.063 (2)	0.7405 (12)	0.153 (7)	0.345 (8)
H1B	1.001248	−0.137151	0.766192	0.229*	0.345 (8)
C1B	0.9053 (17)	0.0324 (15)	0.6486 (10)	0.133 (6)	0.345 (8)
H1BA	0.850989	−0.012521	0.622232	0.160*	0.345 (8)
C2B	1.0239 (15)	0.0430 (19)	0.5902 (14)	0.186 (9)	0.345 (8)
H2BA	1.003882	0.122132	0.534881	0.280*	0.345 (8)
H2BB	1.044114	−0.029514	0.569942	0.280*	0.345 (8)
H2BC	1.101692	0.043478	0.627413	0.280*	0.345 (8)
C3B	0.8059 (16)	0.1510 (13)	0.6445 (11)	0.149 (6)	0.345 (8)
H3BA	0.724542	0.126930	0.674673	0.223*	0.345 (8)
H3BB	0.782508	0.207177	0.578759	0.223*	0.345 (8)
H3BC	0.846576	0.195344	0.677535	0.223*	0.345 (8)

### Atomic displacement parameters (Å^2^)

**Table:**

	*U*^11^	*U*^22^	*U*^33^	*U*^12^	*U*^13^	*U*^23^
F1	0.1060 (13)	0.0556 (11)	0.0826 (12)	−0.0242 (10)	−0.0315 (9)	−0.0220 (10)
O1	0.1174 (18)	0.0798 (18)	0.0625 (14)	−0.0149 (14)	−0.0320 (12)	−0.0096 (13)
O2	0.0827 (14)	0.0481 (14)	0.0633 (12)	−0.0152 (11)	−0.0102 (10)	−0.0050 (11)
N1	0.112 (2)	0.069 (2)	0.0580 (16)	−0.0174 (16)	−0.0288 (14)	−0.0085 (15)
N2	0.0910 (18)	0.0633 (18)	0.0559 (16)	−0.0016 (14)	−0.0208 (13)	−0.0184 (14)
N3	0.0655 (14)	0.0434 (14)	0.0499 (13)	−0.0095 (11)	−0.0098 (11)	−0.0157 (11)
N4	0.0632 (14)	0.0502 (15)	0.0447 (13)	−0.0128 (11)	−0.0068 (10)	−0.0108 (11)
C1	0.091 (2)	0.062 (2)	0.062 (2)	−0.0046 (18)	−0.0144 (16)	−0.0248 (18)
C2	0.0696 (18)	0.053 (2)	0.0481 (17)	−0.0020 (15)	−0.0063 (13)	−0.0145 (16)
C3	0.0642 (17)	0.054 (2)	0.0531 (17)	−0.0091 (14)	−0.0077 (13)	−0.0236 (16)
C4	0.0611 (17)	0.0470 (18)	0.0469 (16)	−0.0073 (14)	−0.0036 (12)	−0.0163 (14)
C5	0.0604 (17)	0.0467 (19)	0.0480 (16)	−0.0082 (14)	0.0028 (12)	−0.0149 (14)
C6	0.0686 (18)	0.0418 (17)	0.0515 (16)	−0.0150 (14)	−0.0021 (13)	−0.0121 (14)
C7	0.0675 (18)	0.0500 (19)	0.0555 (17)	−0.0198 (15)	−0.0057 (14)	−0.0231 (15)
C8	0.0623 (17)	0.0444 (17)	0.0460 (15)	−0.0127 (13)	0.0006 (12)	−0.0154 (13)
C9	0.0632 (16)	0.0378 (16)	0.0494 (15)	−0.0109 (13)	−0.0001 (12)	−0.0178 (13)
C10	0.0565 (16)	0.0434 (17)	0.0429 (15)	−0.0092 (13)	−0.0009 (12)	−0.0153 (13)
C11	0.0564 (16)	0.0463 (18)	0.0440 (15)	−0.0091 (13)	−0.0003 (12)	−0.0153 (13)
C12	0.0619 (19)	0.071 (2)	0.0582 (18)	−0.0141 (15)	−0.0036 (13)	−0.0164 (16)
C13	0.070 (2)	0.087 (3)	0.0651 (19)	−0.0174 (18)	−0.0105 (15)	−0.0177 (19)
C14	0.079 (2)	0.085 (3)	0.063 (2)	−0.0060 (19)	−0.0141 (15)	−0.0062 (19)
C15	0.083 (2)	0.070 (2)	0.0539 (18)	−0.0086 (17)	−0.0047 (14)	−0.0109 (16)
C16	0.0689 (18)	0.0499 (19)	0.0557 (17)	−0.0096 (14)	−0.0011 (13)	−0.0137 (15)
C17	0.0751 (19)	0.0438 (18)	0.0613 (18)	−0.0197 (15)	−0.0098 (14)	−0.0169 (15)
C18	0.084 (2)	0.0416 (18)	0.0646 (19)	−0.0140 (16)	−0.0119 (15)	−0.0262 (16)
C19	0.100 (3)	0.059 (2)	0.084 (2)	−0.004 (2)	−0.0161 (18)	−0.022 (2)
C20	0.122 (3)	0.060 (3)	0.129 (4)	0.009 (2)	−0.041 (3)	−0.037 (3)
C21	0.122 (4)	0.093 (4)	0.170 (5)	0.009 (3)	−0.014 (4)	−0.080 (4)
C22	0.205 (5)	0.103 (4)	0.141 (4)	0.009 (4)	0.052 (4)	−0.064 (4)
C23	0.171 (4)	0.075 (3)	0.090 (3)	0.009 (3)	0.024 (3)	−0.034 (2)
C24	0.089 (2)	0.102 (3)	0.056 (2)	−0.007 (2)	−0.0193 (16)	−0.025 (2)
C25	0.123 (3)	0.094 (3)	0.135 (3)	0.024 (2)	−0.065 (3)	−0.065 (3)
C26	0.129 (3)	0.099 (3)	0.114 (3)	0.014 (2)	−0.046 (2)	−0.061 (3)
C27	0.105 (3)	0.118 (4)	0.069 (2)	−0.029 (2)	−0.0087 (19)	−0.039 (2)
C28	0.167 (4)	0.101 (3)	0.095 (3)	0.037 (3)	−0.069 (3)	−0.043 (3)
C29	0.164 (4)	0.088 (3)	0.097 (3)	0.034 (3)	−0.071 (3)	−0.045 (2)
C30	0.137 (3)	0.133 (4)	0.091 (3)	−0.022 (3)	−0.033 (2)	−0.055 (3)
O1A	0.094 (3)	0.058 (3)	0.106 (4)	−0.009 (3)	0.010 (2)	−0.020 (3)
C1A	0.115 (6)	0.090 (5)	0.122 (6)	−0.021 (4)	0.019 (3)	−0.005 (4)
C2A	0.195 (9)	0.196 (10)	0.123 (6)	−0.062 (8)	−0.024 (5)	0.015 (6)
C3A	0.225 (9)	0.146 (7)	0.215 (9)	−0.118 (7)	0.015 (7)	−0.028 (6)
O1B	0.182 (14)	0.119 (9)	0.081 (7)	0.048 (7)	0.009 (6)	0.013 (7)
C1B	0.142 (10)	0.101 (8)	0.083 (8)	0.015 (8)	0.027 (6)	0.031 (7)
C2B	0.128 (11)	0.186 (14)	0.144 (11)	−0.005 (9)	0.040 (9)	0.042 (10)
C3B	0.176 (12)	0.091 (9)	0.115 (10)	0.021 (7)	−0.002 (8)	0.013 (7)

### Geometric parameters (Å, º)

**Table:**

F1—C7	1.361 (3)	C20—C21	1.334 (6)
O1—N1	1.427 (3)	C21—H21	0.9300
O1—C1	1.333 (4)	C21—C22	1.355 (6)
O2—C5	1.245 (3)	C22—H22	0.9300
N1—C2	1.293 (4)	C22—C23	1.388 (6)
N2—C1	1.292 (4)	C23—H23	0.9300
N2—C2	1.383 (4)	C24—H24	0.9800
N3—C3	1.344 (3)	C24—C25	1.494 (5)
N3—C10	1.399 (3)	C24—C29	1.485 (5)
N3—C17	1.469 (3)	C25—H25A	0.9700
N4—C8	1.393 (3)	C25—H25B	0.9700
N4—C12	1.474 (3)	C25—C26	1.506 (5)
N4—C16	1.463 (3)	C26—H26A	0.9700
C1—C24	1.501 (4)	C26—H26B	0.9700
C2—C4	1.462 (4)	C26—C27	1.464 (5)
C3—H3	0.9300	C27—H27	0.9800
C3—C4	1.363 (4)	C27—C28	1.481 (5)
C4—C5	1.439 (4)	C27—C30	1.515 (5)
C5—C11	1.462 (4)	C28—H28A	0.9700
C6—H6	0.9300	C28—H28B	0.9700
C6—C7	1.351 (3)	C28—C29	1.523 (5)
C6—C11	1.395 (4)	C29—H29A	0.9700
C7—C8	1.417 (4)	C29—H29B	0.9700
C8—C9	1.390 (4)	C30—H30A	0.9600
C9—H9	0.9300	C30—H30B	0.9600
C9—C10	1.395 (3)	C30—H30C	0.9600
C10—C11	1.394 (4)	O1A—H1A	0.8200
C12—H12A	0.9700	O1A—C1A	1.438 (8)
C12—H12B	0.9700	C1A—H1AA	0.9800
C12—C13	1.507 (4)	C1A—C2A	1.489 (9)
C13—H13A	0.9700	C1A—C3A	1.448 (10)
C13—H13B	0.9700	C2A—H2AA	0.9600
C13—C14	1.506 (4)	C2A—H2AB	0.9600
C14—H14A	0.9700	C2A—H2AC	0.9600
C14—H14B	0.9700	C3A—H3AA	0.9600
C14—C15	1.512 (4)	C3A—H3AB	0.9600
C15—H15A	0.9700	C3A—H3AC	0.9600
C15—H15B	0.9700	O1B—H1B	0.8200
C15—C16	1.499 (4)	O1B—C1B	1.430 (10)
C16—H16A	0.9700	C1B—H1BA	0.9800
C16—H16B	0.9700	C1B—C2B	1.460 (13)
C17—H17A	0.9700	C1B—C3B	1.487 (13)
C17—H17B	0.9700	C2B—H2BA	0.9600
C17—C18	1.504 (4)	C2B—H2BB	0.9600
C18—C19	1.362 (4)	C2B—H2BC	0.9600
C18—C23	1.363 (5)	C3B—H3BA	0.9600
C19—H19	0.9300	C3B—H3BB	0.9600
C19—C20	1.377 (5)	C3B—H3BC	0.9600
C20—H20	0.9300		
			
C1—O1—N1	106.8 (2)	C20—C21—C22	119.9 (5)
C2—N1—O1	102.7 (3)	C22—C21—H21	120.1
C1—N2—C2	103.3 (3)	C21—C22—H22	119.9
C3—N3—C10	119.1 (2)	C21—C22—C23	120.2 (4)
C3—N3—C17	119.8 (2)	C23—C22—H22	119.9
C10—N3—C17	121.1 (2)	C18—C23—C22	120.4 (4)
C8—N4—C12	116.85 (19)	C18—C23—H23	119.8
C8—N4—C16	116.8 (2)	C22—C23—H23	119.8
C16—N4—C12	111.4 (2)	C1—C24—H24	106.9
O1—C1—C24	118.7 (3)	C25—C24—C1	110.4 (3)
N2—C1—O1	112.6 (3)	C25—C24—H24	106.9
N2—C1—C24	128.7 (3)	C29—C24—C1	113.8 (3)
N1—C2—N2	114.5 (3)	C29—C24—H24	106.9
N1—C2—C4	124.0 (3)	C29—C24—C25	111.5 (3)
N2—C2—C4	121.6 (3)	C24—C25—H25A	109.0
N3—C3—H3	117.4	C24—C25—H25B	109.0
N3—C3—C4	125.2 (3)	C24—C25—C26	113.1 (3)
C4—C3—H3	117.4	H25A—C25—H25B	107.8
C3—C4—C2	118.2 (3)	C26—C25—H25A	109.0
C3—C4—C5	119.5 (2)	C26—C25—H25B	109.0
C5—C4—C2	122.3 (3)	C25—C26—H26A	109.0
O2—C5—C4	124.3 (2)	C25—C26—H26B	109.0
O2—C5—C11	120.9 (3)	H26A—C26—H26B	107.8
C4—C5—C11	114.8 (2)	C27—C26—C25	112.9 (3)
C7—C6—H6	119.8	C27—C26—H26A	109.0
C7—C6—C11	120.4 (3)	C27—C26—H26B	109.0
C11—C6—H6	119.8	C26—C27—H27	106.9
F1—C7—C8	118.6 (2)	C26—C27—C28	110.4 (3)
C6—C7—F1	117.8 (3)	C26—C27—C30	112.9 (4)
C6—C7—C8	123.6 (3)	C28—C27—H27	106.9
N4—C8—C7	121.0 (3)	C28—C27—C30	112.4 (3)
C9—C8—N4	123.7 (2)	C30—C27—H27	106.9
C9—C8—C7	115.3 (2)	C27—C28—H28A	108.8
C8—C9—H9	119.0	C27—C28—H28B	108.8
C8—C9—C10	122.0 (3)	C27—C28—C29	113.7 (3)
C10—C9—H9	119.0	H28A—C28—H28B	107.7
C9—C10—N3	120.6 (2)	C29—C28—H28A	108.8
C11—C10—N3	118.9 (2)	C29—C28—H28B	108.8
C11—C10—C9	120.5 (3)	C24—C29—C28	112.5 (3)
C6—C11—C5	119.6 (2)	C24—C29—H29A	109.1
C10—C11—C5	122.2 (3)	C24—C29—H29B	109.1
C10—C11—C6	118.1 (2)	C28—C29—H29A	109.1
N4—C12—H12A	109.4	C28—C29—H29B	109.1
N4—C12—H12B	109.4	H29A—C29—H29B	107.8
N4—C12—C13	111.1 (2)	C27—C30—H30A	109.5
H12A—C12—H12B	108.0	C27—C30—H30B	109.5
C13—C12—H12A	109.4	C27—C30—H30C	109.5
C13—C12—H12B	109.4	H30A—C30—H30B	109.5
C12—C13—H13A	109.5	H30A—C30—H30C	109.5
C12—C13—H13B	109.5	H30B—C30—H30C	109.5
H13A—C13—H13B	108.1	C1A—O1A—H1A	111.0
C14—C13—C12	110.8 (3)	O1A—C1A—H1AA	112.1
C14—C13—H13A	109.5	O1A—C1A—C2A	102.5 (8)
C14—C13—H13B	109.5	O1A—C1A—C3A	109.0 (8)
C13—C14—H14A	109.8	C2A—C1A—H1AA	112.1
C13—C14—H14B	109.8	C3A—C1A—H1AA	112.1
C13—C14—C15	109.4 (3)	C3A—C1A—C2A	108.4 (9)
H14A—C14—H14B	108.2	C1A—C2A—H2AA	109.5
C15—C14—H14A	109.8	C1A—C2A—H2AB	109.5
C15—C14—H14B	109.8	C1A—C2A—H2AC	109.5
C14—C15—H15A	109.1	H2AA—C2A—H2AB	109.5
C14—C15—H15B	109.1	H2AA—C2A—H2AC	109.5
H15A—C15—H15B	107.8	H2AB—C2A—H2AC	109.5
C16—C15—C14	112.5 (2)	C1A—C3A—H3AA	109.5
C16—C15—H15A	109.1	C1A—C3A—H3AB	109.5
C16—C15—H15B	109.1	C1A—C3A—H3AC	109.5
N4—C16—C15	111.7 (3)	H3AA—C3A—H3AB	109.5
N4—C16—H16A	109.3	H3AA—C3A—H3AC	109.5
N4—C16—H16B	109.3	H3AB—C3A—H3AC	109.5
C15—C16—H16A	109.3	C1B—O1B—H1B	141.8
C15—C16—H16B	109.3	O1B—C1B—H1BA	103.5
H16A—C16—H16B	107.9	O1B—C1B—C2B	105.0 (17)
N3—C17—H17A	108.6	O1B—C1B—C3B	118.6 (16)
N3—C17—H17B	108.6	C2B—C1B—H1BA	103.5
N3—C17—C18	114.6 (2)	C2B—C1B—C3B	120.3 (15)
H17A—C17—H17B	107.6	C3B—C1B—H1BA	103.5
C18—C17—H17A	108.6	C1B—C2B—H2BA	109.5
C18—C17—H17B	108.6	C1B—C2B—H2BB	109.5
C19—C18—C17	119.8 (3)	C1B—C2B—H2BC	109.5
C19—C18—C23	117.8 (3)	H2BA—C2B—H2BB	109.5
C23—C18—C17	122.4 (3)	H2BA—C2B—H2BC	109.5
C18—C19—H19	119.2	H2BB—C2B—H2BC	109.5
C18—C19—C20	121.6 (4)	C1B—C3B—H3BA	109.5
C20—C19—H19	119.2	C1B—C3B—H3BB	109.5
C19—C20—H20	119.9	C1B—C3B—H3BC	109.5
C21—C20—C19	120.1 (4)	H3BA—C3B—H3BB	109.5
C21—C20—H20	119.9	H3BA—C3B—H3BC	109.5
C20—C21—H21	120.1	H3BB—C3B—H3BC	109.5
			
F1—C7—C8—N4	−3.4 (4)	C7—C6—C11—C5	−179.3 (2)
F1—C7—C8—C9	173.7 (2)	C7—C6—C11—C10	1.4 (4)
O1—N1—C2—N2	−0.7 (3)	C7—C8—C9—C10	1.8 (4)
O1—N1—C2—C4	178.5 (2)	C8—N4—C12—C13	164.6 (3)
O1—C1—C24—C25	110.5 (4)	C8—N4—C16—C15	−166.8 (2)
O1—C1—C24—C29	−15.7 (5)	C8—C9—C10—N3	−179.0 (2)
O2—C5—C11—C6	−2.0 (4)	C8—C9—C10—C11	1.7 (4)
O2—C5—C11—C10	177.2 (2)	C9—C10—C11—C5	177.4 (2)
N1—O1—C1—N2	−0.3 (4)	C9—C10—C11—C6	−3.3 (4)
N1—O1—C1—C24	−178.4 (3)	C10—N3—C3—C4	−2.6 (4)
N1—C2—C4—C3	178.0 (3)	C10—N3—C17—C18	−76.8 (3)
N1—C2—C4—C5	−1.8 (4)	C11—C6—C7—F1	−175.2 (2)
N2—C1—C24—C25	−67.3 (5)	C11—C6—C7—C8	2.4 (4)
N2—C1—C24—C29	166.5 (3)	C12—N4—C8—C7	−53.5 (3)
N2—C2—C4—C3	−2.8 (4)	C12—N4—C8—C9	129.7 (3)
N2—C2—C4—C5	177.4 (2)	C12—N4—C16—C15	55.4 (3)
N3—C3—C4—C2	178.2 (2)	C12—C13—C14—C15	−55.0 (4)
N3—C3—C4—C5	−2.0 (4)	C13—C14—C15—C16	53.5 (4)
N3—C10—C11—C5	−1.9 (4)	C14—C15—C16—N4	−54.0 (4)
N3—C10—C11—C6	177.3 (2)	C16—N4—C8—C7	171.0 (2)
N3—C17—C18—C19	146.5 (3)	C16—N4—C8—C9	−5.8 (4)
N3—C17—C18—C23	−35.2 (4)	C16—N4—C12—C13	−57.6 (3)
N4—C8—C9—C10	178.8 (2)	C17—N3—C3—C4	176.8 (2)
N4—C12—C13—C14	57.9 (3)	C17—N3—C10—C9	5.8 (4)
C1—O1—N1—C2	0.6 (3)	C17—N3—C10—C11	−174.9 (2)
C1—N2—C2—N1	0.6 (4)	C17—C18—C19—C20	177.4 (3)
C1—N2—C2—C4	−178.7 (3)	C17—C18—C23—C22	−178.3 (4)
C1—C24—C25—C26	−178.1 (4)	C18—C19—C20—C21	1.3 (6)
C1—C24—C29—C28	174.5 (4)	C19—C18—C23—C22	0.1 (6)
C2—N2—C1—O1	−0.1 (3)	C19—C20—C21—C22	−0.5 (7)
C2—N2—C1—C24	177.7 (3)	C20—C21—C22—C23	−0.5 (8)
C2—C4—C5—O2	4.5 (4)	C21—C22—C23—C18	0.7 (8)
C2—C4—C5—C11	−175.9 (2)	C23—C18—C19—C20	−1.0 (5)
C3—N3—C10—C9	−174.9 (2)	C24—C25—C26—C27	54.2 (5)
C3—N3—C10—C11	4.4 (4)	C25—C24—C29—C28	48.9 (5)
C3—N3—C17—C18	103.8 (3)	C25—C26—C27—C28	−54.2 (5)
C3—C4—C5—O2	−175.3 (2)	C25—C26—C27—C30	179.0 (4)
C3—C4—C5—C11	4.3 (4)	C26—C27—C28—C29	53.2 (5)
C4—C5—C11—C6	178.3 (2)	C27—C28—C29—C24	−51.5 (6)
C4—C5—C11—C10	−2.4 (3)	C29—C24—C25—C26	−50.6 (5)
C6—C7—C8—N4	179.0 (2)	C30—C27—C28—C29	−179.7 (4)
C6—C7—C8—C9	−3.9 (4)		

### Hydrogen-bond geometry (Å, º)

**Table:**

*D*—H···*A*	*D*—H	H···*A*	*D*···*A*	*D*—H···*A*
C12—H12*A*···F1	0.97	2.19	2.873 (4)	126
C16—H16*B*···O1*A*^i^	0.97	2.53	3.441 (9)	155
C17—H17*B*···O1*A*^i^	0.97	2.59	3.504 (9)	157
C17—H17*B*···O1*B*^i^	0.97	2.55	3.491 (17)	162
O1*A*—H1*A*···O2^ii^	0.82	2.09	2.906 (9)	180
O1*B*—H1*B*···O2^ii^	0.82	1.84	2.662 (19)	180

Symmetry codes: (i) −*x*+1, −*y*, −*z*+2; (ii) *x*+1, *y*−1, *z*.

### Hydrogen-bond characteristics

**Table:**

D—H···A	D—H, Å	H···A, Å	D···A, Å	D—H···A, deg
C12—H12A···F1	0.97	2.19	2.873 (4)	126.4
C16—H16B···O1Ai	0.97	2.53	3.440 (8)	155.3
C17—H17B···O1Ai	0.97	2.59	3.503 (9)	157.1
C17—H17B···O1Bi	0.97	2.55	3.491 (18)	162.4
O1A—H1A···O2ii	0.82	2.09	2.905 (8)	179.7
O1B—H1B···O2ii	0.82	1.84	2.662 (19)	179.6

Symmetry codes: (i) -*x* + 1, -*y*, -*z* + 2; (ii) *x* - 1, *y* + 1, *z*.
